# Natural History of Scoliosis in Children with NF1: An Observation Study

**DOI:** 10.3390/healthcare9070881

**Published:** 2021-07-13

**Authors:** Giuseppe Toro, Claudia Santoro, Daniele Ambrosio, Giovanni Landi, Martina Scilipoti, Antimo Moretti, Marco Paoletta, Sara Liguori, Alfredo Schiavone Panni, Stefania Picariello, Giovanni Iolascon

**Affiliations:** 1 Department of Medical and Surgical Specialties and Dentistry, University of Campania “Luigi Vanvitelli”, 80138 Naples, Italy; giuseppe.toro@unicampania.it (G.T.); danieleambrosio15@gmail.com (D.A.); g.landi17@libero.it (G.L.); antimo.moretti@unicampania.it (A.M.); sara.liguori@unicampania.it (S.L.); alfredo.schiavonepanni@unicampania.it (A.S.P.); giovanni.iolascon@gmail.com (G.I.); 2 Department of Clinical Sciences and Translational Medicine, University of Rome Tor Vergata, 00133 Rome, Italy; 3 Department of Woman, Child and of General and Specialised Surgery, University of Campania “Luigi Vanvitelli”, 80138 Naples, Italy; claudia.santoro@unicampania.it (C.S.); scilipoti.martina@gmail.com (M.S.); stefaniapicariello34@gmail.com (S.P.); 4 Department of Mental Health, Physical and Preventive Medicine, University of Campania “Luigi Vanvitelli”, 80138 Naples, Italy

**Keywords:** neurofibromatosis type 1, scoliosis, treatment, natural history, dystrophic, non-dystrophic

## Abstract

(1) Background. Scoliosis is the most common musculoskeletal manifestation of Neurofibromatosis type 1 (NF1), and it might be dystrophic (D) or non-dystrophic (ND) depending on the presence of dysplastic changes of the spine. The aim of our study was to describe the characteristics and natural history of patients with NF1 and scoliosis. (2) Methods. We retrospectively reviewed records from patients with NF1 and scoliosis. Scoliosis was classified as D if at least two dystrophic changes were documented at imaging. (3) Results. Of the 438 patients reviewed, 43 fulfilled inclusion criteria; 17 were classified in D group and 26 in ND. The groups did not differ in age and localization of scoliosis curvature. Surgery was needed more often in D group, but the between-group difference was not significant. Male-to-female ratios of 3:1 and 4:1 were reported in surgically treated NF1 patients with ND and D scoliosis, respectively. (4) Conclusions. Our data suggests independently by the presence of dysplastic changes affecting the spine that males with NF1 are more often affected by scoliosis that requires surgery.

## 1. Introduction

Neurofibromatosis type 1 (NF1) is one of the most common inherited neurological disorders with a prevalence of 1/3000 individuals [1]. It is caused by heterozygous mutations in the NF1 gene located on 17q11.2 chromosome region. This gene encodes for the neurofibromin protein, a multifunctional regulatory protein of several physiological processes (i.e., regulation of cell proliferation and differentiation through the RAS/MAPK and RAS/PI3K/AKT signal transduction pathways; regulation of the cell cycle progression interfering with the cAMP/protein kinase A pathways; regulation of the intracellular transport) [2]. 

NF1 is phenotypically manifested as a disorder of neural crest cells, making patients prone to develop benign tumors of both central and peripheral nervous systems. Skin hyperpigmentation, as well as the café-au-lait spots and freckles, are peculiar findings of NF1. Diagnosis is still based on clinical criteria set up by the National Institutes of Health (NIH) in 1987 [3]. 

Bone dysplasia is one of abovementioned criteria, and both flat (for example, the sphenoid) or long bones (typically tibia, but not exclusively) might be affected.

However, scoliosis is the most common skeletal manifestation in these patients. It is estimated that approximately 2% of all pediatric scoliosis is due to NF1 [4].

Patients with NF1 can develop either dystrophic or non-dystrophic scoliosis based on the occurrence of vertebral dysplasia (i.e., vertebral scalloping, paravertebral neurofibromas, dural ectasia, rib penciling) in the former. Scoliosis in NF1 is more commonly non-dystrophic, and these cases, theoretically, should overlap with idiopathic scoliosis in terms of natural history [3]. Dystrophic scoliosis instead tends to evolve more rapidly into severe curves requiring a surgical approach.

Moreover, some dysplastic changes (i.e., dural ectasia, rib penciling) are likely to induce a higher indication for surgery [5,6,7].

Very few reports on the characterization of scoliosis in children with NF1 had been published; most of them before the emanation of both NIH criteria for NF1 diagnosis and the standardization of scoliosis taxonomy by the Scoliosis Research Society (SRS) [8,9,10]. In this context, our retrospective study was designed to provide new data for the definition of (1) the prevalence of dystrophic scoliosis; (2) the age at onset and other demographic data of scoliosis in NF1 and (3) management of scoliosis in NF1 and the characteristics of surgically treated patients.

## 2. Materials and Methods

We retrospectively reviewed clinical notes of 438 children referred to the NF1 care unit of the University of Campania “Luigi Vanvitelli” between 1992 and 2017. The NF care unit is composed of a multidisciplinary team (two pediatricians, two orthopedists, one physiatrist, one neuroradiologist, one oculist and one neurosurgeon) with expertise on NF1. The pediatrician is the case manager responsible for performing the first screening evaluation, coordinating the team activities and defining the NF1 diagnosis. The diagnosis of NF1 is established according to the NIH criteria [3]. The pediatrician organizes the team schedule, and after the first clinical assessment, each patient is evaluated by team specialists for different clinical issues. Patients are routinely screened at least once a year by the multidisciplinary team. Based on orthopedist and physiatrist evaluation, spine x-rays are prescribed at the onset of new spinal deformities (i.e., scoliosis or hyperkyphosis) or in case of their progression. The presence of radiographic vertebral dystrophic changes is routinely reported through a pre-established form. Magnetic resonance imaging (MRI) evaluation is performed in case of neurological impairment, a clear discordance between clinical and radiographic findings or when surgical procedures are needed. For this study, we selected patients with NF1 diagnosis according to NIH criteria and diagnosis of scoliosis, defined as a curve >10° on the coronal plane [11]. We collected for these the following data: demographic data, inheritance type of NF1, both age and curve degree at diagnosis of scoliosis, curve progression at the end of follow-up, the type of scoliosis treatment and any associated musculoskeletal deformities.

Patients with incomplete clinical or radiological history were excluded. 

Scoliosis was classified according to the location of the apical vertebra/disc and defined as “dystrophic” when at least two signs of vertebral dystrophy were found in X-rays and/or MRI (Table 1) [5,7,12]. Patients were then divided in two groups: “dystrophic” and “not dystrophic”. Included patients were divided into three categories according to age at onset of scoliosis (infantile, juvenile, adolescent) and in two super categories (“early onset” and “late onset” scoliosis) according to the SRS terminology [11,13].

Statistical analysis was performed using the *t*-test for continuous data and the chi-square or Fisher’s exact test for categorical data. Significance was set at *p* < 0.05.

## 3. Results

### 3.1. Prevalence of Dystrophic Scoliosis in NF1

**Figure 1:**
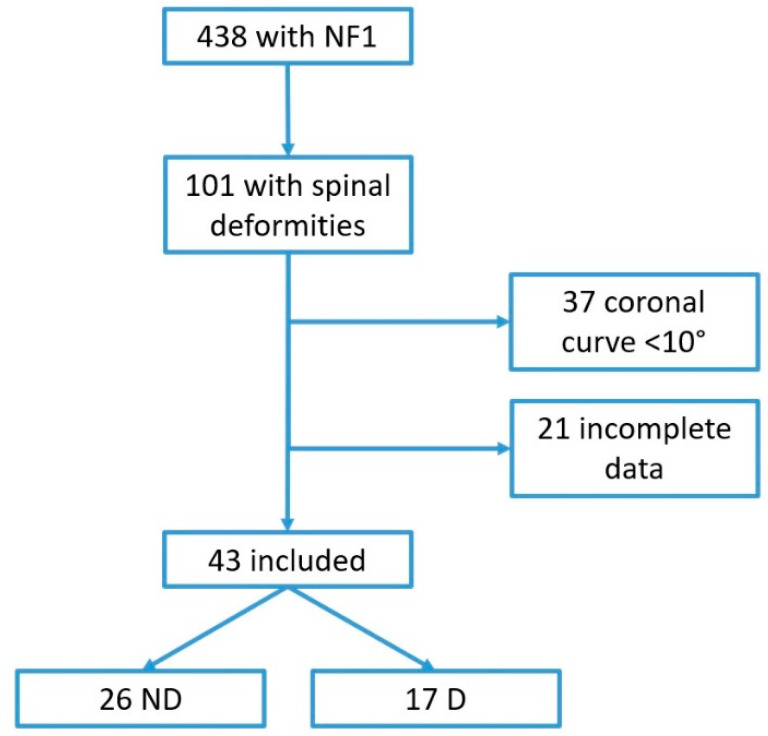
Flowchart of population selection.

Among 438 clinical reports reviewed, we identified 101 NF1 children (0–18 years) with a spinal deformity. A total of 43 patients were included (24 males, 19 females; M:F = 1.3:1) (see Figure 1), accounting for 9.8% of the entire pediatric cohort. MRI was performed in 22/43 patients to complete the diagnosis of dystrophic scoliosis that was reported in five cases. Table 2 summarizes all data collected for both dystrophic and non-dystrophic groups. 

### 3.2. Age at Onset and Other Demographic Data of Scoliosis 

**Figure 2:**
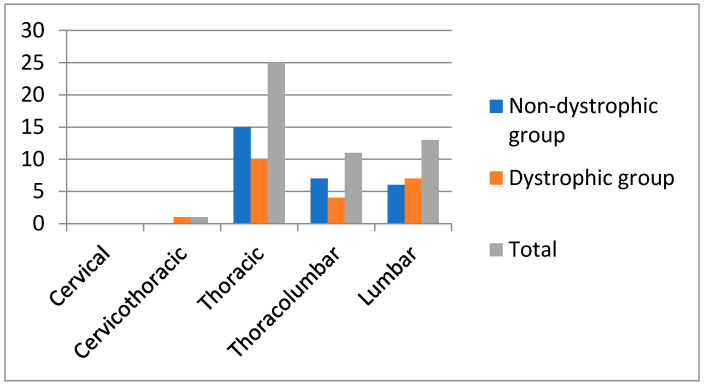
Distribution of scoliotic curves in the two groups of patients.

The mean age at diagnosis of scoliosis was 9.4 years (range 2.4–16.8). Among the 43 patients included, 50 curves were identified. In both groups, the right thoracic represented the most frequent curve (Figure 2). Although the curve side distribution between groups was apparently different in the thoracolumbar tract, this difference was not statistically significant (*p* = 0.242).

The two groups did not differ in sex distribution. Mean age at diagnosis of scoliosis in dystrophic group was lower compared with non-dystrophic group (8.8 vs. 10.8 years), and the percentage of “early onset” scoliosis was higher in dystrophic group (12/17, 70%) than non-dystrophic group (13/26, 50%), without statistically significant differences. 

Most of patients in the dystrophic group (10/17, 59%) presented an inherited form of NF1, with a higher prevalence of maternal forms (7/17). 

Among dystrophic changes, paravertebral neurofibromas were the most observed, followed by vertebral scalloping (Table 2). Dural sac anomalies were found in seven patients in the dystrophic group, such as ectasia (five), syringomyelia (one), and Chiari I malformation (one). A short segmental curve was observed even in 61% (16/26) of patients of the non-dystrophic group. 

### 3.3. Management of Scoliosis and Characteristics of Surgically Treated Patients

A total of 18 patients (11 non-dystrophic, 7 dystrophic) of the entire cohort were conservatively treated using a brace, whereas 9 required surgery (4 non-dystrophic, 5 dystrophic) (Table 2).

The percentage of surgically treated patients was higher in the dystrophic group (15% non-dystrophic vs. 29% dystrophic).

In the non-dystrophic group, the mean age at diagnosis of scoliosis of the four surgically treated patients (15%) was 9.5 years (range 5–13) with a male-female ratio of 3:1. Two patients were affected by thoracic scoliosis, whereas two were affected by double curves (thoracic and thoracolumbar). Finally, two of the surgically treated patients in the non-dystrophic group were previously treated with a brace (two Lyonnaise, one Milwaukee). In the dystrophic group, the mean age at diagnosis of scoliosis of the five surgically treated patients (29%) was 7 years (range 3–11) and the male-to-female ratio was 4:1. In dystrophic group, two of these five patients received bracing (one Milwaukee, one Cheneau) before surgery (see Table 2). The two groups of surgically treated patients did not differ in age and sex.

## 4. Discussion

### 4.1. Prevalence of Dystrophic Scoliosis in NF1

The exact prevalence of scoliosis in NF1 is unknown and extremely variable (2–69%) [4,14], mostly depending on differences in study design and assessed patients. In our cohort of 438 children with NF1, we found a prevalence of scoliosis of 9.8%. This data was similar to that previously reported by Akbarnia et al. in 1992 (10%) [15] but lower than that recently observed by Lykissas et al. (19%) [7]. The higher prevalence reported by these latter authors might be linked to their expertise in surgical treatment of vertebral abnormalities.

Patients with NF1 may develop dystrophic or non-dystrophic scoliosis [12]. In our population, dystrophic scoliosis occurred in approximately 39% of patients. This percentage was quite higher than reported in the available literature (ranging from 10 to 30%) [15,16]. A possible explanation is that it may be related to the high percentage of patients who underwent to MRI in our population. MRI should be considered an important imaging modality in the evaluation of patients with scoliosis, considering that it was demonstrated to be able to identify intraspinal abnormalities also in presumed idiopathic scoliosis [17]. These observations are even more relevant in NF1. In fact, dystrophic changes were observed in more than one-third of patients previously classified as “non-dystrophic” in the series by Ramachandran et al. [18].

Pathogenesis of different scoliosis forms observed in NF1 is still unclear and probably multifactorial. Some authors hypothesized that the presence of tumors as well as para/intracanal spine deformities might play a role in this context (i.e., paraspinal neurofibromas or dural ectasia) [19]. Other authors supported a putative role of abnormal control of both osteoclastic and osteoblastic activity. Particularly, Rhodes et al. postulated an enhanced bone resorptive activity of NF1 haploinsufficient hematopoietic derived osteoclasts both in long bones and vertebrae [20,21], whereas Wu et al. hypothesized impaired osteoblasts differentiation in patients with NF1 [22]. 

### 4.2. Age at Onset and Other Demographic Data of Scoliosis 

**Figure 3:**
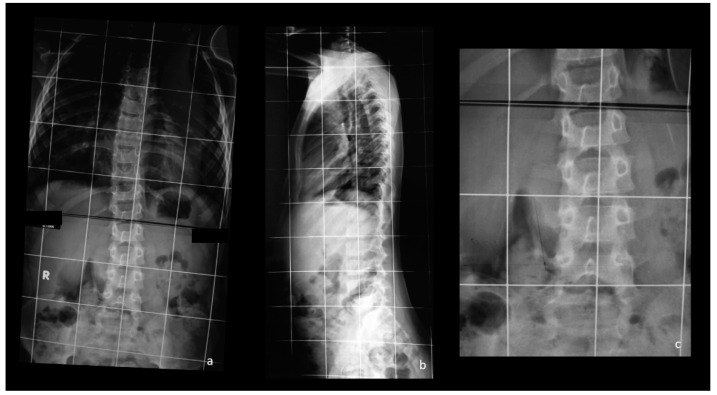
Anteroposterior (**a**) and latero-lateral (**b**) views of a lumbar dystrophic early onset scoliosis. Note the dysplastic pedicles of the lumbar spine, the intervertebral foraminal widening in (**b**). In (**c**), a detail of the anteroposterior view. Note the apparent overlapping of bone between the vertebrae on the concave side, lately confirmed by MRI to be paravertebral neurofibromas.

Dystrophic scoliosis is mainly characterized by vertebral dysplasia, and some of the other dysplastic changes (i.e., dural ectasia, rib penciling) are associated with a higher risk for surgery [5,6,7]. In our series, the most frequently observed dystrophic features were vertebral scalloping and paravertebral neurofibromas (16% and 22%, respectively). Ramachandran et al. observed that, although these latter findings could be observed in both types of scoliosis in NF1, these lesions were more commonly located on the concave side of the curve in the dystrophic population [18]. Moreover, paraspinal neurofibromas were associated with a more severe vertebral deformity [18]. In our cohort, paravertebral neurofibromas were observed in 11 patients with dystrophic and in 3 patients with non-dystrophic scoliosis, and patients in dystrophic group presented a higher burden of paraspinal neurofibromas compared with patients in non-dystrophic group. In Figure 3, an example of a dystrophic scoliosis with dysplastic pedicles and paravertebral neurofibromas is shown.

**Figure 4:**
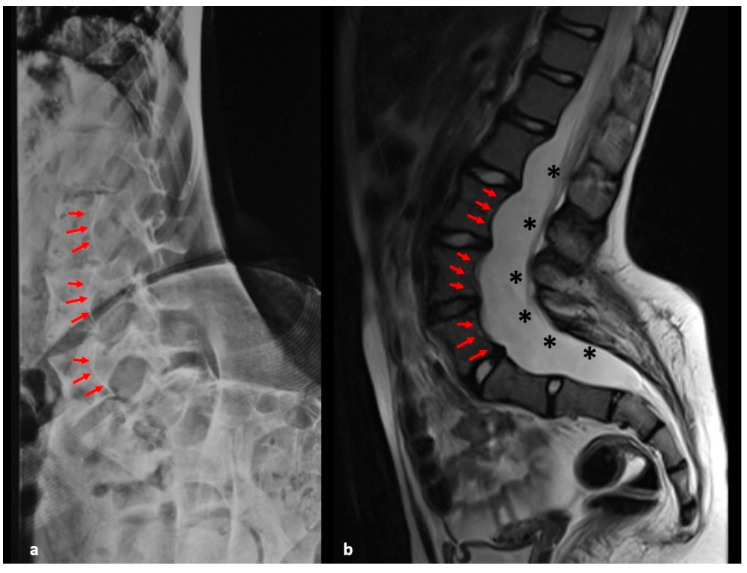
X-ray (**a**) and MRI (**b**) of the lumbar spine of a child (F) with NF1 and dystrophic scoliosis. Note the vertebral scalloping (arrows) and the dural ectasia (asterisks).

Dural ectasia is described as an expansion of the dural sac. Numerous authors hypothesized that dural ectasia erodes the vertebra, causing posterior vertebral scalloping and leading to spinal deformity, subluxation or vertebral dislocation [12]. The pathogenesis of dural ectasia in NF1 is still unclear, and it could be also associated with other genetic disorders, particularly collagen disorders, namely Ehlers–Danlos and Marfan syndromes [23]. Intriguingly, NF1 might play a role in collagen gene expression in fibroblasts [24]. We observed cooccurrence of dural ectasia and spinal deformity in 11% of patients with scoliosis. The incidence grew up to 29% in those with dystrophic scoliosis. Figure 4 shows an example of cooccurrence of dural ectasia and spinal deformity in a patient with a dystrophic scoliosis.

Cooccurrence of multiple signs of vertebral dysplasia are predictors of curve evolution [5,7], while both vertebral scalloping and dural ectasia, as well as the costal penciling, are predictive factors of corrective surgery [5,7].

Our observation of a high percentage (57%) of short-range curves also in non-dystrophic scoliosis is in line with Akbarnia et al. [15]. Therefore, in our opinion, and contrary to what is believed, the short segmental curve is not a key dystrophic feature [5,7,25,26].

In our population, dystrophic scoliosis tended to occur earlier than non-dystrophic. However, the between-group difference was not statistically significant. 

Interestingly, we reported a high prevalence of males among NF1 patients with scoliosis (M:F = 1.3:1), in particular in those surgically treated (M:F = 3:1 non-dystrophic surgically treated; M:F = 4:1 dystrophic surgically treated). Male-to-female ratio was in line to that observed by Lykissas et al. [7] (M:F = 1:0.7, regardless the type of scoliosis), on a cohort of over 120 NF1 patients with scoliosis. Sex distribution of our population was exceptionally different from that reported in an idiopathic scoliosis population with a similar geographical origin [27]. In fact, Aulisa et al. reported a M:F ratio 1:3.3 on a population of 181 patients with idiopathic scoliosis, [27]. This ratio decreased up to M:F = 1:4 in scoliosis with a magnitude higher than 30° (namely those with a higher risk of surgery) [27]. This latter observation was surprising, especially considering that non-dystrophic population characteristics and natural history have been generally considered similar to those of idiopathic scoliosis. The higher percentage of males among NF1 patients with scoliosis might be partly related to the previous observation of a higher number of cases of atypical scoliosis (namely, those more frequently associated with intraspinal abnormalities such as left thoracic, left thoracic/right lumbar, left thoracic/right thoracolumbar, right and left double thoracic, long right thoracic (King IV) and right and left triple and quadruple curve patterns) in males compared to females [28]. However, although the number of dystrophic features were different between the two groups, in the present study, we did not observe a significant difference in sex distribution. Therefore, some unsolved questions around the different sex distribution between patients with idiopathic scoliosis and those with NF1 still remain. However, in our opinion, our results may suggest male sex as a main risk factor for NF1 scoliosis and for surgical indication, also considering the higher incidence of behavioral and emotional problems reported in males with NF1 that could further worsen patients’ compliance to conservative treatment [29].

### 4.3. Management of Scoliosis and Characteristics of Surgically Treated Patients

The differentiation of dystrophic from non-dystrophic scoliosis should be carefully defined, considering their supposed different natural history. Indeed, theoretically, a larger number of patients with dystrophic scoliosis require surgery [19]. However, in our population, any difference in percentage of surgery was not observed between the two groups. This result has fundamental impact on prognosis of these patients; to note, the three of four non-dystrophic patients who had undergone surgery were previously conservatively treated. Considering the total number of patients in non-dystrophic group receiving bracing (11), the percentage of patients subsequently surgically treated is exceptionally high (27%) compared to the general population (about 14%), as reported by Rigo et al. [30]. This remarkable difference in brace treatment efficacy might be explained by challenges in patients’ compliance in non-dystrophic scoliosis, which might be further compromised by the occurrence of cognitive impairment, including attention disorders and emotional, psychological and social issues in NF1 patients compared to the general population [1]. 

To the best of our knowledge, no data about the efficacy of conservative treatment in non-dystrophic scoliosis compared with idiopathic scoliosis were available.

Among patients with dystrophic scoliosis requiring surgery, three showed associated deformities such as dural ectasia (two) and Chiari type I malformation. This latter abnormality has been already reported in patients with NF1 [31], but its correlation with the scoliosis onset had been recently questioned by Strahle et al. [32]. The observation of a Chiari I malformation in a patient with scoliosis has a remarkable effect on the surgical treatment, often requiring a prophylactic decompression.

### 4.4. Limitations and Strengths of the Study

To the best of our knowledge, the present is the second largest study on scoliosis in patients with NF1 defined using both NIH criteria and scoliosis taxonomy proposed by SRS. Our study had some limitations. First, the retrospective design and the lack of direct assessment of radiographic and MRI images could lead to underestimating the incidence of vertebral dystrophies; however, the patient’s approach through a skilled multidisciplinary team and standardized records allowed us to be very confident with the quality of the presented data. Another limitation was the small sample size and the lack of multiple comparison corrections that might had underpowered the statistical analysis; however, the strict inclusion criteria were necessary to reduce sampling errors. Moreover, genetic data are lacking, thus limiting an adequate genotype/phenotype analysis that might affect musculoskeletal manifestations in NF1 patients. A further limitation is the lack of additional signs of NF1 reported in our records that may lead to selection bias. However, the criteria used for the diagnosis of NF1 and our multidisciplinary approach with a prompt follow-up protocol make us confident that our cohort is representative of a population affected by NF1.

## 5. Conclusions

Scoliosis is the most common skeletal manifestation observed in NF1. Apparently, male patients are more often affected by scoliosis in NF1 compared to the idiopathic scoliosis population. Natural history of dystrophic scoliosis differs from that of non-dystrophic with a higher risk for corrective surgery. On the other hand, non-dystrophic scoliosis in NF1 is not always responsive to bracing. Therefore, all NF1 patients need a careful and systematic follow-up made by an experienced team to early recognize developing curves and to improve appropriateness of management strategies in order to gain better outcomes and improve patients’ compliance. Further studies are required to better understand the efficacy of conservative treatment in non-dystrophic scoliosis and the role of sex in the incidence and evolution of scoliosis in NF1.

## Figures and Tables

**Table 1:** Criteria for the definition of a dystrophic scoliosis [6,7,12].

Vertebral Dystrophic Changes
Vertebral Scalloping (present when the depth of scalloping is >3 mm in the thoracic spine or >4 mm in the lumbar spine)
Rib penciling (rib width lower than the narrowest portion of the second rib)
Spindling of the transverse processes (loss of 50% from the height of the transverse process measured in the middle point between the lateral edge of the vertebral body and tip of the transverse process and compared with the contralateral normal side or uninvolved vertebra above or below)
Focal, short-segmented curve (involving 6 or less vertebrae)
Dural ectasia
Paraspinal tumors or plexiform neurofibromas close to the scoliosis curve
Vertebral wedging
Intervertebral foraminal widening
Widened interpediculate distances
Dysplastic pedicles

Note: Criteria are rated as “yes/no”. At least two dystrophic changes must be found at X-rays and/or MRI to define dystrophic scoliosis.

**Table 2:** Characteristics of the two study groups. **ND:** non-dystrophic **D:** dystrophic.

	NF1 Cohort	ND Group (*n* = 26)	D Group (*n* = 17)	*p*-Value
**Demographic data**				
Mean age at scoliosis diagnosis (yrs)	N/A	10.08 (2–12)	8.8 (3–16)	0.167
Sex (M)	246	14	9	1
Early Onset (%)	N/A	13/26 (50%)	12/17 (70%)	0.307
Infantile scoliosis		0	1	0.395
Juvenile scoliosis		13	11	0.369
Adolescent scoliosis		13	5	0.219
Sporadic NF1	215	18	7	0.114
Hereditary NF1	223	8	10	
Maternal NF1	115	6	7	1
Paternal NF1	90	2	3	
Unspecified	18			
**Dystrophic features**	N/A			
Vertebral scalloping (*n*)		0	10	0.001
Dural ectasia (*n*)		0	5	0.006
Paravertebral neurofibromas (*n*. of patients)		3	11	0.0006
Cervicothoracic (*n*. of patients)		1	4	1
Thoracic (*n*. of patients)		0	5	0.51
Thoracolumbar (*n*. of patients)		0	3	1
Lumbar (*n*. of patients)		2	3	0.17
Focal, short segment curve, *n* (%)		16 (57%)	13 (59%)	0.34
Syringomyelia (*n*)		0	1	
Chiari I (*n*)		0	1	
Long bone dysplasia (*n*)		1	0	
**Treatment**	N/A			
Conservative treatment, *n* (%)		11 (42%)	7 (41%)	0.94
Cheneau		3	3	
Lyonnaise		3	2	
Boston		2	1	
Milwaukee		3	1	
Surgery (*n*)	N/A	4 (15%)	5 (29%)	0.4

Legend: Infantile scoliosis: age at diagnosis <3 years; Juvenile scoliosis: age at diagnosis 3–9 years; Adolescent scoliosis: age at diagnosis >10 years; Conservative treatment: braces; N/A: not appli-cable.

## Data Availability

The data presented in this study are available on request from the corresponding author.

## References

[1] Wang Z., Liu Y. (2010). Research Update and Recent Developments in the Management of Scoliosis in Neurofibromatosis Type 1. Orthopedics.

[2] Abramowicz A., Gos M. (2015). Neurofibromin—Protein structure and cellular functions in the context of neurofibromatosis type I pathogenesis. Postępy Hig. Med. Dośw..

[3] (1988). National Institutes of Health Consensus Development Conference Statement: Neurofibromatosis.

[4] Vitale M.G., Guha A., Skaggs D.L. (2002). Orthopaedic Manifestations of Neurofibromatosis in Children: An Update. Clin. Orthop. Relat. Res..

[5] Durrani A.A., Crawford A.H., Chouhdry S.N., Saifuddin A., Morley T.R. (2000). Modulation of Spinal Deformities in Patients with Neurofibromatosis Type 1. Spine.

[6] Funasaki H., Winter R.B., Lonstein J.B., Denis F. (1994). Pathophysiology of spinal deformities in neurofibromatosis. An analysis of seventy-one patients who had curves associated with dystrophic changes. J. Bone Jt. Surg. Am. Vol..

[7] Lykissas M.G., Schorry E.K., Crawford A.H., Gaines S., Rieley M., Jain V.V. (2013). Does the Presence of Dystrophic Features in Patients with Type 1 Neurofibromatosis and Spinal Deformities Increase the Risk of Surgery?. Spine.

[8] Mccarroll H.R. (1950). Clinical Manifestations of Congenital Neurofibromatosis. J. Bone Jt. Surg. Am..

[9] Rezaian S.M. (1976). The Incidence of Scoliosis Due to Neurofibromatosis. Acta Orthop. Scand..

[10] Winter R.B., Moe J.H., Bradford D.S., Lonstein J.E., Pedras C.V., Weber A.H. (1979). Spine deformity in neurofibromatosis. A review of one hundred and two patients. J. Bone Jt. Surg. Am. Vol..

[11] Scoliosis Research Society SRS Revised Glossary. https://www.srs.org/professionals/online-education-and-resources/glossary/revised-glossary-of-terms.

[12] Tsirikos A.I., Saifuddin A., Noordeen M.H. (2005). Spinal deformity in neurofibromatosis type-1: Diagnosis and treatment. Eur. Spine J..

[13] Scoliosis Research Society SRS Patients and Families Early Onset Scoliosis. https://www.srs.org/patients-and-families/conditions-and-treatments/parents/scoliosis/early-onset-scoliosis.

[14] Gutmann D., Ferner R.E., Listernick R.H., Korf B.R., Wolters P.L., Johnson K.J. (2017). Neurofibromatosis type 1. Nat. Rev. Dis. Prim..

[15] Akbarnia B.A., Gabriel K.R., Beckman E., Chalk D. (1992). Prevalence of Scoliosis in Neurofibromatosis. Spine.

[16] Crawford A.H., Lykissas M.G., Schorry E.K., Gaines S., Jain V., Greggi T., Viskochil D. (2012). Neurofibromatosis: Etiology, Commonly Encountered Spinal Deformities, Common Complications and Pitfalls of Surgical Treatment. Spine Deform..

[17] Rajasekaran S., Kamath V., Kiran R., Shetty A. (2010). Intraspinal anomalies in scoliosis: An MRI analysis of 177 consecutive scoliosis patients. Indian J. Orthop..

[18] Ramachandran M., Tsirikos A.I., Lee J., Saifuddin A. (2004). Whole-Spine Magnetic Resonance Imaging in Patients with Neurofibromatosis Type 1 and Spinal Deformity. J. Spinal Disord. Tech..

[19] Feldman D.S., Jordan C., Fonseca L. (2010). Orthopaedic Manifestations of Neurofibromatosis Type 1. J. Am. Acad. Orthop. Surg..

[20] Rhodes S.D., Zhang W., Yang D., Yang H., Chen S., Wu X., Li X., Yang X., Mohammad K.S., Guise T.A. (2015). Dystrophic Spinal Deformities in a Neurofibromatosis Type 1 Murine Model. PLoS ONE.

[21] Zhang W., Rhodes S.D., Zhao L., He Y., Zhang Y., Shen Y., Yang D., Wu X., Li X., Yang X. (2011). Primary osteopathy of vertebrae in a neurofibromatosis type 1 murine model. Bone.

[22] Wu X., Estwick S.A., Chen S., Yu M., Ming W., Nebesio T.D., Li Y., Yuan J., Kapur R., Ingram D. (2006). Neurofibromin plays a critical role in modulating osteoblast differentiation of mesenchymal stem/progenitor cells. Hum. Mol. Genet..

[23] Böker T., Vanem T.T., Pripp A.H., Rand-Hendriksen S., Paus B., Smith H.-J., Lundby R. (2019). Dural ectasia in Marfan syndrome and other hereditary connective tissue disorders: A 10-year follow-up study. Spine J..

[24] Nehls M.C., Grapilon M.L., Brenner D.A. (1992). NF-I/Sp1 Switch Elements Regulate Collagen α1(I) Gene Expression. DNA Cell Biol..

[25] Parisini P., Di Silvestre M., Greggi T., Paderni S., Cervellati S., Savini R. (1999). Surgical Correction of Dystrophic Spinal Curves in Neurofibromatosis. Spine.

[26] Wilde P.H., Upadhyay S.S., Leong J.C.Y. (1994). Deterioration of Operative Correction in Dystrophic Spinal Neurofibromatosis. Spine.

[27] Aulisa A.G., Giordano M., Guzzanti V., Falciglia F., Pizzetti P., Toniolo R.M. (2019). Effectiveness of school scoliosis screening and the importance of this method in measures to reduce morbidity in an Italian territory. J. Pediatr. Orthop. B.

[28] Wang W., Zhu Z., Zhu F., Sun C., Wang Z., Sun X., Qiu Y. (2012). Different Curve Pattern and Other Radiographical Characteristics in Male and Female Patients with Adolescent Idiopathic Scoliosis. Spine.

[29] Rietman A.B., Van Der Vaart T., Plasschaert E., Nicholson B., Oostenbrink R., Krab L.C., Descheemaeker M.-J., Wit M.-C.Y., Moll H.A., Legius E. (2018). Emotional and behavioral problems in children and adolescents with neurofibromatosis type 1. Am. J. Med Genet. Part B Neuropsychiatr. Genet..

[30] Rigo M., Reiter C., Weiss H.-R. (2003). Effect of conservative management on the prevalence of surgery in patients with adolescent idiopathic scoliosis. Pediatr. Rehabil..

[31] Miraglia E., Fabbrini G., Di Biasi C., Iacovino C., Ferrazzano G., Gualdi G., Calvieri S., Giustini S. (2016). Chiari type 1 malformation in Neurofibromatosis type 1: Experience of a center and review of the literature. La Clin. Ter..

[32] Strahle J., Smith B.W., Martinez M., Bapuraj J.R., Muraszko K.M., Garton H.J.L., Maher C.O. (2015). The association between Chiari malformation Type I, spinal syrinx, and scoliosis. J. Neurosurg. Pediatr..

